# Electric Cell-Substrate Impedance Sensing (ECIS) with Microelectrode Arrays for Investigation of Cancer Cell – Fibroblasts Interaction

**DOI:** 10.1371/journal.pone.0153813

**Published:** 2016-04-18

**Authors:** Trong Binh Tran, Changyoon Baek, Junhong Min

**Affiliations:** School of Integrative Engineering, Chung-Ang University, Heukseok-dong, Dongjak-gu, Seoul, Republic of Korea; Chunag-Ang University, REPUBLIC OF KOREA

## Abstract

The tumor microenvironment, including stromal cells, surrounding blood vessels and extracellular matrix components, has been defined as a crucial factor that influences the proliferation, drug-resistance, invasion and metastasis of malignant epithelial cells. Among other factors, the communications and interaction between cancer cells and stromal cells have been reported to play pivotal roles in cancer promotion and progression. To investigate these relationships, an on-chip co-culture model was developed to study the cellular interaction between A549—human lung carcinoma cells and MRC-5—human lung epithelial cells in both normal proliferation and treatment conditions. In brief, a co-culture device consisting of 2 individual fluidic chambers in parallel, which were separated by a 100 μm fence was utilized for cell patterning. Microelectrodes arrays were installed within each chamber including electrodes at various distances away from the confrontation line for the electrochemical impedimetric sensing assessment of cell-to-cell influence. After the fence was removed and cell-to-cell contact occurred, by evaluating the impedance signal responses representing cell condition and behavior, both direct and indirect cell-to-cell interactions through conditioned media were investigated. The impact of specific distances that lead to different influences of fibroblast cells on cancer cells in the co-culture environment was also defined.

## Introduction

There is growing evidence demonstrating that the tumor microenvironment, including stromal cells, inflammatory cells, extracellular matrix (ECM), cytokines, vessels and growth factors, plays an important role in the initiation, progression and invasion of cancer [1–3]. During tumorigenesis, cancer cells interact dynamically with surrounding stromal cells, such as fibroblasts, adipose cells and resident immune cells. Among these, fibroblasts form the largest group of stromal cells and appear to function prominently in cancer progression [4–5].

First described in the late 19^th^ century, fibroblasts are elongated, non-vascular, non-epithelial and non-inflammatory cells of the connective tissue with extended cell processes that show a fusiform or spindle-like shape in profile. Fibroblasts perform many important functions, including the deposition of ECM, the regulation of epithelial differentiation, and the regulation of inflammation; they are also involved in wound healing [5]. During normal proliferation in healthy organs, fibroblasts synthesize and secrete various types of collagens (i.e., types I, III, and V) as well as fibronectin and proteoglycans, which are the essential constituents of ECM [6]. Fibroblasts also secrete type IV collagen and laminin, which assist in the formation of the basement membrane [7]. In wounded organs, fibroblasts play an important role in the healing process by invading lesions and generating ECM to serve as a scaffold for other cells [8].

In the early stage of tumorigenesis, cancer cells form a neoplastic lesion within the boundary of the basement membrane but separated from the surrounding tissue [9]. The basement membrane, fibroblasts, immune cells, capillaries and ECM surrounding the cancer cells form an area that is called the tumor microenvironment. As the principle source of ECM components, fibroblasts are defined as a key cellular component of tumors. In association with cancer cells, normal fibroblasts can acquire a perpetually activated phenotype by direct cell-cell communication or by various stimuli that arise when tissue injury occurs [10]. Activated fibroblasts exhibit the up-regulations of ECM-degrading matrix metalloproteinases-2, 3 and 9 (MMP-2, MMP-3 and MMP-9) as well as many growth factors, which induce proliferative signals to adjacent epithelial cells [11]. From this close association, a question arises about the heterotypic cellular interactions between tumor cells and fibroblasts in the tumor microenvironment. In the past decade, a number of research studies have clarified the effect of fibroblasts on various aspects of cancer cell behavior including proliferation, angiogenesis, invasion, metastasis and drug resistance; however, cancer cells behavior has yet to be completely explained. Prominently, Stoker et al. (1966), Wadlow et al. (2009) and Flaberg et al. (2011, 2012) have shown that normal fibroblasts can inhibit the growth of cancer cells *in vitro* and they termed this effect as neighbor suppression [12–15]. Flaberg et al. (2012) designed a co-culture assay with H2A-mRFP-labeled tumor cells on a mono-layer of fibroblasts [15]. Over the course of 62.5 h, tumor cells proliferation and motility were significantly inhibited by the fibroblasts through direct cell-to-cell interaction. To fully understand these effects, we conjectured whether there is an indirect neighbor interaction between fibroblasts and cancer cells, which we termed as a distance effect. The given hypothesis is that the inhibitory effect of fibroblasts on cancer cells is a function of the distance between these 2 cell types in a common stromal microenvironment.

**Fig 1:**
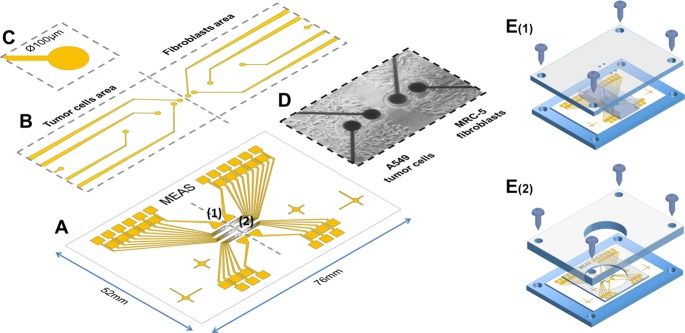
(A) Microelectrode arrays (1: working electrode, 2: common counter electrode) were fabricated on experimental glass slides (76mm x 52mm) by common photolithography processes. SU-8 photoresist was used as passivation layer to cover the conducting traces. (B) The sensing platform was divided into 2 areas, one for the cancer cells and one for microenvironment agents. Several mirco-sized working electrodes were installed in each area at different distances: 100, 250, 650, 1450 and 3050μm far from the confrontation line. (C) A single electrode was 100μm in diameter. (D) An image of a real chip after the co-culturing of 2 different cell types on 2 sides. (E) The co-culture patterning process using a dual-chamber mold. (E_1_) After treating the surface for cell culture, the cell chip was placed on the designed fixture, and the dual-chamber mold was fixed at the proper location by screws. The 2 chambers were separated each other by a 100μm thick wall for cell patterning. (E_2_) After the cells in both chamber had attached to the chip surface, the dual-chamber mold was replaced by a well-type open reservoir. A PDMS bed was also installed to prevent solution from leaking. The cell chip was connected to the measurement system and was kept in the incubator during the measurements.

In this study, we proposed a simple co-culture model with embedded high-throughput microelectrode arrays (MEA) using an electric cell-substrate impedance sensing (ECIS) assay (Fig 1) to monitor tumor cell conditions continuously when confronted with cultured fibroblasts. This electrical sensing method was utilized in this study due to the prominent advantages; this method is non-invasive, simple to setup, easy to perform, extraordinarily sensitive to cellular conditions and capable of real-time monitoring [16,17]. The MEA were patterned on both sides of the cell culture areas at various distances from the separation line. During the cell interactions, each electrode continuously records an impedance signal through a high-throughput data acquisition system. This type of impedimetric data has been acknowledged to reflect cellular adhesion, spreading, proliferation and viability in treatment conditions with environmental toxins, drugs, and chemicals, as well as other substances.

In this study, we showed the inhibitory effect of MRC-5 human lung fibroblasts on the normal proliferation of A549 human lung cancer cells. Impedance signal differences were determined to indirectly demonstrate the distance effect between MRC-5 and A549 cells in a co-culture environment. Particularly in the drug treatment conditions, we also observed an intensive suppression in direct fibroblast—cancer interaction, which was higher than that exerted by indirect interaction. Additionally, the inhibitory effect of fibroblasts was confirmed using the fluorescence-activated cell sorting (FACS) method in a mixed culture model. Our study was aimed to mimic an *in-vivo* therapeutic stromal effect on tumor progression with a novel detection and analysis technique in the real-time domain.

## Materials and Methods

### MEA fabrication

The MEA pattern was fabricated on glass microscope slides (52 mm × 76 mm) by common photolithography processes using AZ-5214E (MicroChemicals GmbH, Germany) as a negative photoresist for a lift-off process. The patterning was followed by the deposition of a 250 Å/500 Å Ti/Au bi-layer by using an e-beam evaporation system, and the unneeded portions were selective removed in acetone. After the electrodes were formed, we performed a second photolithography step to apply a 2 μm thick layer of SU-8 2002 (MicroChem, USA) as an isolation layer for the gold traces, leaving only the electrodes arrays and contact pads expodes.

### Cell culture

MRC-5 human lung fibroblasts and A549 human lung cancer cells were obtained from the American Type Culture Collection (ATCC, Manassas, VA, USA) and were cultured in RPMI medium 1640 (Gibco) supplemented with 10% Fetal bovine serum (FBS, Gibco) and 1% antibiotics/antimycotics (Gibco). These cells were maintained in condition of 37°C and of 5% CO_2_ and sub-cultured under treatment of trypsin-EDTA (Gibco) solution. The number of cells was counted using the Cellometer (Cellometer Auto T4, USA).

### Cell patterning on MEA chip

In this study, the cell was patterned using a common physical barrier method to create cell-free regions for collective cell migration [18]. A physical barrier was applied prior to cell seeding to block cell mixing, and the removal of the physical barrier initiates the cell migration and confrontation process. Acrylic fixtures for the cell chip and dual-chamber mold were designed using AutoCAD and were fabricated using a CNC milling machine (Fig 1E). Before being used for cell culture, all of the devices were sterilized in an alcohol bath and were dried in an oven with UV light. After the dual-chamber mold was fixed to the cell chip in the proper position using screws, each chamber was treated with a collagen solution (10%) for 15 min at room temperature to promote cell adhesion. Then, the chambers were washed with 1X PBS, and the 2 types of cell suspension (5×10^5^ cells/chamber) were injected into the 2 separate chambers. After 12 h, when both cell types had attached well and had formed a monolayer on the chip surface, the chambers were disassembled and were replaced by the well-type reservoir. The cell chip was connected to the ECIS system and kept in the incubator during the measurements.

### CellTracker staining for migration checking

MRC-5 fibroblasts were stained with CellTracker before co-culture to assess their migration into the area of A549 tumor cells. In brief, MRC-5 cells were cultured in a cell culture dish (SPL), and were harvested using Trypsin-EDTA (Gibco). The cells were centrifuged, the supernatant was removed, and the cells were re-suspended in 1 ml of culture media (RPMI-1640 with 10% FBS, 1% PS, 25 mM HEPES, Gibco). The cell solution received 20 μM of CellTracker^TM^ (Life Technologies, CellTracker^TM^ Green CMFDA) and the cells were subsequently incubated for 30 min. Then, the cells were centrifuged and supernatant was removed; then, the cells were re-suspended in 1 ml of fresh culture media. The stained cells were then ready to be injected into the co-culture chamber as described in the cell patterning section. The cell staining was confirmed using fluorescence microscopy (Eclipse 80i, Nikon).

### Flow cytometry analysis

A549 and MRC-5 cells (5×10^5^ cells/ml) were cultured in 6-well plates for both the mono-culture of A549 cells and the co-culture of A549 and MRC-5 cells). Each cell type was treated with 30 μM of curcumin (Sigma-Aldrich) for 3 days. Then, the cells were washed three times with cold PBS (Sigma-Aldrich) and suspended binding buffer (10 mM HEPES, 140 mM NaCl, 2.5 mM CaCl_2_, pH 7.4, Sigma-Aldrich). Each sample received 5 μM Annexin V conjugated Alexa Fluor® 594 (Life Technologies, excitation/emission: 590/617 nm) and was incubated for 15 min at room temperature. The cells were analyzed using an Accuri C6 flow cytometer (BD Bioscience) and the data were analyzed with FlowJo software (Treestar).

### MTS assay

Curcumin (Sigma-Aldrich) was prepared in DMSO and diluted to desired concentrations in cell culture media. For the experiment, cells were cultivated at 1×10^4^ cells/100 μl of the initial seeding amount in a 96-well cell culture plate for 24 h. The A549 cells and MCR-5 fibroblasts were treated with various concentrations of curcumin (1, 10, 30, 50, 70, 100 and 500 μM) for 3 days. The Cell Counting Kit-8 solution (Dojindo Inc.) was diluted to 1:10 with medium and added to each well of the plate, and then the cells were incubated for 4 h. Cell proliferation was measured at 450 nm using a Wallac 1420 VICTOR3 V microplate reader.

### Measurement system

An Agilent 4284A Impedance Precision Meter (Agilent CA, USA) and a designed switching relay circuit were operated to simultaneously measure the impedance of the electrodes arrays. The measuring conditions were 10 mV and 10 kHz at 5 min intervals. LabVIEW (National Instrument, TX, USA) software was used to control the meter and record numerical data to a PC, and then the data were automatically plotted on a real-time chart.

### Statistical analysis

All the data were expressed as the means (±SD) of 3 repetitions. Statistical analysis was performed by a one-way factorial ANOVA, followed by the Tukey’s honestly significant difference (HSD) test of difference. Differences were considered to be statistically significant at p values less than 0.05.

## Results

### MEA characterization via monocultures of tumor cells and fibroblasts

**Fig 2:**
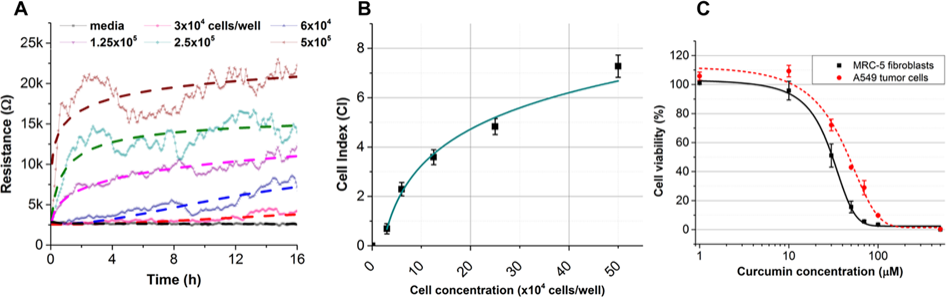
(A) Resistance responses of MEA to various concentrations of A549 tumor cells in monoculture for 16 h (at 10mV, 10kHz). (B) This calibration curve represents the correlation between A549 cell density and cell index value. (C) MTS results from of monocultures of A549 cells and MRC-5 fibroblasts after 72 h of exposure to curcumin. The IC50 values for the A549 cells and MRC-5 cells were 50 μM and 30 μM, respectively. The data are shown as the mean (±SD) of 3 repetitions.

In the first experimental step, the MEA cell chips were validated via monocultures of A549 tumor cells and MRC-5 fibroblasts in normal proliferative conditions. Before operating the cell measurement system, the arrays were adjusted (by LabVIEW) to normalize the systematic differences, such as wiring, soldering and conductive traces. The normalized cell-free resistance of each electrode was fixed at 2.6 kΩ. Fig 2A shows the resistance responses when single electrodes were challenged by various concentrations of A549 cells. The data obtained over 16 h were fitted by a hill algorithm for a growth/sigmoidal model (OriginPro 9). Below 1.25×10^5^ cells/well, the resistance curves corresponded well to the fitting curves. At higher cell densities, a lag phase appeared from 4 h to 8 h, when the cells were rearranging themselves on binding the site prior to forming a monolayer on the electrodes. To easily estimate viability throughout this study, a dimensionless parameter termed the cell index (CI) was defined as the relative change in measured electrical resistance and can be calculated using the equation below

$$CI=\frac{R_{i}}{R_{cell-free}}-1$$

where R_i_ is the resistance of the cell-covered electrodes, and R_cell-free_ is the resistance of blank electrodes (2.6 kΩ). After 16 h, a calibration curve was plotted, which represented the correlation between CI value and cell seeding density (Fig 2B).

Additionally, A549 tumor cells and MRC-5 fibroblasts were separately treated with various doses of curcumin to define an appropriate treatment condition for the co-culture study. Curcumin has proven effective in inducing apoptosis as well as inhibition of proliferation of lung adenocarcinoma cell line—A549 [19]. In this study, curcumin was chosen to create the treatment environment for the proposed co-culture model. An MTS assay was performed, and a cell viability curve was plotted after 72 h (Fig 2C). The results showed that curcumin has a significant toxic effect on both tumor cells and fibroblasts at 10 μM. The IC50 values for the A549 cells and MRC-5 cells were 50 μM and 30 μM, respectively. The treatment dose at 30 μM was chosen for the subsequent co-culture experiment, where tumor cells were simultaneously exposed to both the toxic effect of curcumin and the inhibitory effect of adjacent fibroblasts.

### Inhibitory effect of fibroblasts on tumor cells in mixed culture

Tumor cell proliferation in this type of co-culture model is determined by properties of both the cancer cells and the fibroblasts, which could exert opposing proliferative effects *in vitro*. For example, AG09877 fibroblasts stimulate the proliferation of carcinoma T47D cells, while AG04351 fibroblasts exhibit a growth-inhibition effect on the same cancer cell line [13]. To determine the reciprocal effects between A549 and MRC-5, a FACS assay was performed with a mixed culture model using these 2 cell lines. The detection scheme was based on the differences in phosphatidylserine (PS) location between healthy cells and apoptotic cells. In normal viable cells, PS is located on the cytoplasmic surface of the cell membrane. When the cells start to undergo apoptosis, PS is translocated from the inside to the outside of the cell membrane and is exposed to the external cellular environment. Using a specific phospholipid-binding protein (Annexin V-conjugated Alexa Fluor® 594) and a flow cytometry sorting system, the early-stage apoptotic cells can be distinguished from the whole cell population.

**Fig 3:**
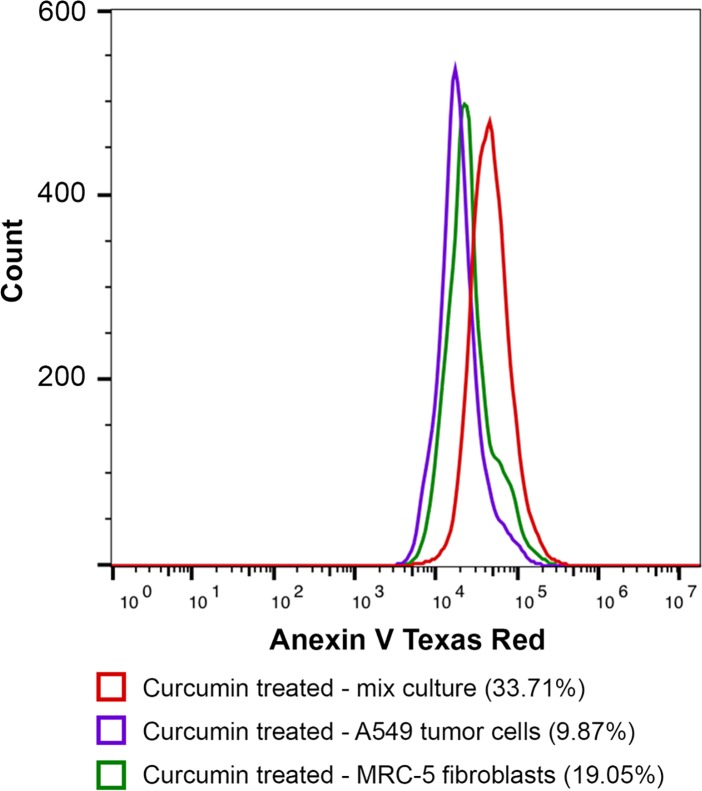
Histogram results of curcumin treatment at 30μM by FACS assay. Different percentages of apoptotic cells were obtained in the A549 tumor cells and MRC-5 fibroblasts monocultures and in the mixed culture of these 2 cell lines. [A color figure can be viewed in the online issue].

As shown in Fig 3, curcumin treatment in the monocultures induced apoptosis in both A549 cells and MRC-5 cells at 9.87% and 19.05% apoptotic cells, respectively. This ratio was less significant than our MTS results as well as the results of other previous studies [20]. These differences were explained by the lack of dead cells, which cannot be discriminated by FACS with only PS staining.

In mixed cultures without a neighbor effect between the tumor cells and fibroblasts, a ratio of total apoptotic cells was expected from 9.87% to 19.05% in the same experimental conditions. Interestingly, the ratio of apoptotic cells in the mixed cultures was determined to be 33.71%. This result suggested that the cell-to-cell inhibitory effect was present in parallel with the toxic effect of curcumin for both cell types. However, specific effects on each cell line were difficult to determine using FACS.

### Inhibitory effect of fibroblasts on the normal proliferation of tumor cells

**Fig 4:**
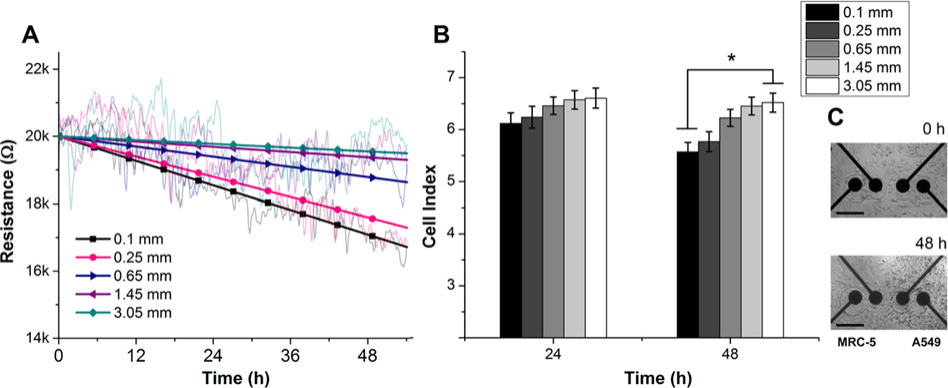
(A) Cellular resistance responses (at 10mV, 10kHz) of A549 tumor cells as a function of time and distance from fibroblasts over 54 h of co-culture in normal growth conditions. The raw data were linearly-fitted and converted to CI value with respect to the cell-free electrode response (2.6 kΩ). (B) CI values of tumor cells at 5 different distances away from the cultured fibroblasts. After 48 h in co-culture, the CI values indicated that the tumor cells that were confronted with fibroblasts were clearly inhibited in comparison with distant cells. (*p < 0.02). (C) Bright-field images of the confrontation between tumor cells and fibroblasts at 0 h and 48 h. The scale bar represents 200 μm.

To discriminate the particular contribution of each cell line to the total inhibitory effect, we examined cellular conditions via impedance responses in a co-culture model. Raw resistance data of A549 cells at various distances away from fibroblasts (0.1, 0.25, 0.65, 1.45, and 3.05 mm) were linearly fitted and normalized, as shown in Fig 4A. The corresponding resistance data after 24 h and 48 h were converted into CI values using the equation shown in section 1 (Fig 4B). During the first 24 h after cell contact, the distance effect was indistinct, and the CI values did not show significant differences between the 1^st^ electrode array (0.1 mm) and the 5^th^ electrode array (3.05 mm).

A significant difference in the proliferation of A549 cells was observed at 48 h. The CI values of A549 cells in directly contact and adjacently contact with fibroblasts (i.e., at the 1^st^ and 2^nd^ electrode arrays) slightly decreased from 6.12 and 6.23 to 5.57 and 5.77, while those of the further A549 cells (i.e., at 3^rd^, 4^th^ and 5^th^ electrode arrays) maintained stable impedance responses at the confluent cell density (Fig 4C). These result clearly showed that fibroblasts are able to inhibit the proliferative rate of tumor cells in confronted cultures more effectively than effects mediated through the media environment.

### Fibroblasts–tumor cells interaction in treatment conditions: obvious neighbor effect over distances

**Fig 5:**
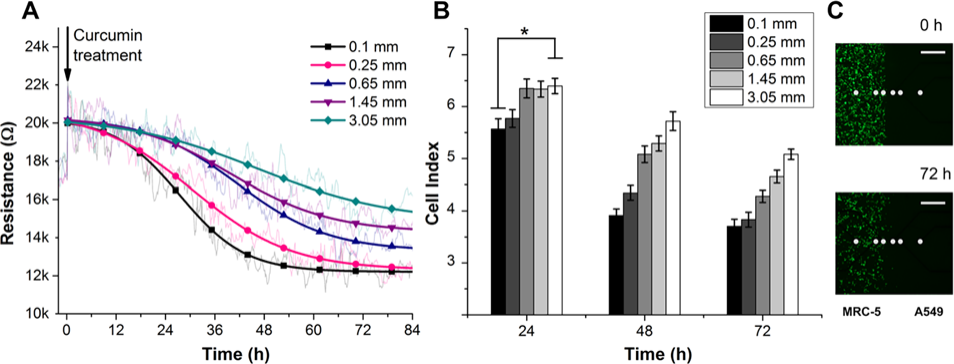
(A) Cellular resistance responses (at 10mV, 10kHz) of A549 tumor cells as a function of time and distance from fibroblasts over 84 h of co-culture in the curcumin treatment condition. The raw data were fitted and converted to CI values with respect to the cell-free electrode responses (2.6 kΩ). (B) CI values of the tumor cells at 5 different distances away from the cultured fibroblasts. The inhibitory effect of fibroblasts on tumor cells was more clearly observed in the toxic environment after 24 h (*p <0.04). (C) The migration rate of fibroblasts into the tumor cell area was assessed using a cell tracker staining assay. In the treatment condition, no significant migration was observed. The gray dots indicate the location of the electrodes arrays. The scale bar represents 500 μm.

To provide a full understanding of the neighbor effect between fibroblasts and tumor cells *in vitro*, we constructed a co-culture model with MRC-5 cells and A549 cells in therapeutic conditions. Curcumin was introduced into the confronted cultures at the previously defined concentration, 30μM; impedance was then monitored for the following 84 h. The average responses were normalized and nonlinearly fitted using a dose-response algorithm (Fig 5A). Interestingly, the distance effect was clearly demonstrated at early times after drug exposure (Fig 5A and 5B). After 24 h, the average CI values obtained were 5.57, 5.77, 6.35, 6.34 and 6.40 for tumor cells that were located at 0.1, 0.25, 0.65, 1.45 and 3.05 mm away from the fibroblast confrontation line. After 48 h and 72 h, significant decreases of all the CI values and the appearance of gaps between the arrays clearly reflected the intensive combined apoptotic effect of both the anti-cancer drug activity and growth inhibition via fibroblast interactions. We also performed a migration assay under the confronted co-culture conditions to investigate the migration ability of fibroblasts into the tumor cells area and thus be able to distinguish the direct cell-to-cell interaction effect from the total inhibitory effect (Fig 5C). After 72 h, the remaining fluorescent intensity showed the efficient apoptotic effect of curcumin on the MRC-5 cells, but no significant migration was observed. These results corresponded well with those of other studies about the effects of curcumin treatment on the migration kinematics of cultured fibroblasts.

## Discussion

Impedimetric cell-based biosensors have been acknowledged as a versatile and reliable characterization platform for bio-analytical studies due to their prominent advantages, such as high sensitivity, high throughput and real-time recording capability. Using various geometrical electrode designs, ECIS platforms have been extensively utilized in the studies of cell attachment and spreading [21,22], cell motility [23], cell layer barrier functions [24], as well as for *in vitro* toxicological studies and drug screening [25,26]; these platforms also been integrated with other bio-analytical systems [27]. The outstanding electrode design is the microelectrode array, which consists of many uniform single microelectrodes on one shared platform. Because they are on the order of microns in size, single microelectrodes are able to address the heterogeneous properties of cells that are concealed within average cells populations, instead of providing a collective cellular response to a given physiological condition. However, the use of single microelectrodes in cellular measurement has to address the extraordinary sensitivity of cell behaviors such as micro-movements, attachment, spreading and proliferation. This signal fluctuation presents a challenge for determining actual cell behavior on an electrode surface. The solution provided in this study was using arrays for measurement repetition, and then applying proper fitting methods to the obtained average data. Using this method, the relative changes of cell viability can be shown via CI number and can easily be converted to percentages in comparison with initial CI values.

**Fig 6:**
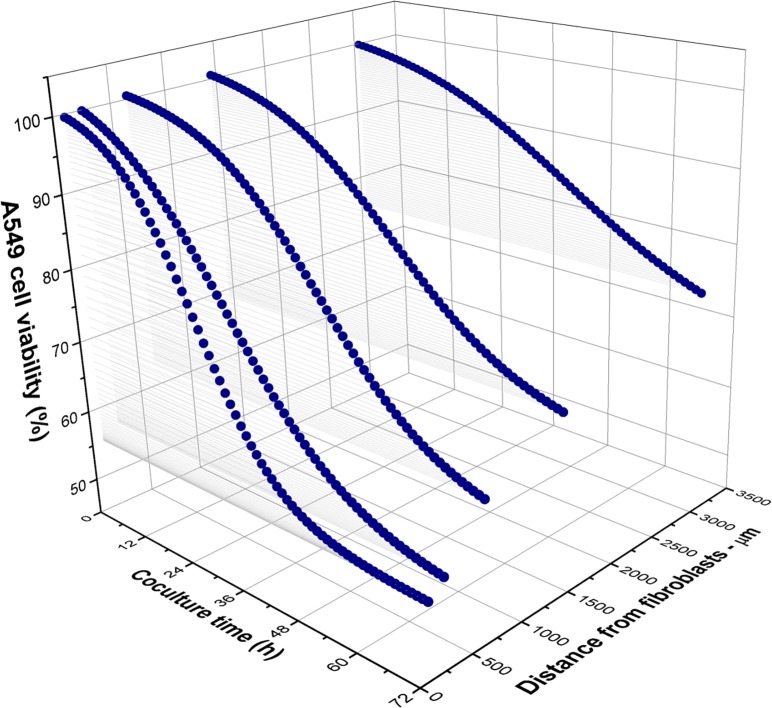
Summary of A549 cell viability as a function of co-culture time and distance from fibroblasts over 72 h in the curcumin treatment condition.

This study was aimed to propose a simple but effective method to investigate the interactions of tumor cells and fibroblasts in a co-culture model, with specific focus on the distance effect between these 2 cell lines. The overall inhibitory and toxic effects on A549 cancer cells are shown in Fig 6. It should be noted that other previous studies have examined the inhibition of tumor cell proliferation and motility by fibroblasts [28–31]; the common conclusion was that the inhibition effect was both contact- and soluble factor-dependent. However, the effective distance between tumor cells and fibroblasts had not yet been determined. By integrating an MEA platform into a co-culture model, we successfully determined the differences of A549 tumor cell behaviors at various distances away from MRC-5 fibroblasts in both normal proliferation and treatment conditions. Our results showed that the influences on the tumor cells could be categorized into 3 different zones based on the viability of the tumor cells. The first zone was located up to 0.25 mm away from the fibroblast confrontation line, where cancer cells were strongly inhibited in both normal and toxic conditions. This result can be explained by the involvement of the surface molecules of fibroblasts as well as the soluble factors that are secreted because of the confrontation between fibroblasts and tumor cells [31]. The second zone was located up to 3 mm away from the first zone, where the effect in normal growth conditions was indistinct but was clearly demonstrated in the treatment condition. This result can also be explained by the soluble factors secreted from fibroblasts from a limited distance. The third zone encompassed the remaining area. In the normal co-cultures, the A549 tumor cells in this zone maintained CI values of 6.6 and 6.5, which indicated a normal proliferation rate despite the cross-talk of distant cells. In the treatment condition, A549 cell viability was determined to be 75.6%, which was approximately the same value determined by the mitochondrial based MTS assay (72.1%). This equivalence obviously indicated that conditioned media did not cause any inhibitory effect when the cancer cells were more than 3 mm away from the fibroblasts.

The response of malignant human cells to the stromal microenvironment and therapeutic regimens is a fundamental topic in cancer research, but a full understanding has not been achieved due to an overreliance on end-point assays [32–34]. In this study, we introduced a well-established assay to a novel sensing platform for the first time to facilitate the continuous monitoring and the location-dependent assessment of cellular inhibition effects. Our results emphasized that the factor of tumor size could affect the efficiency of cancer chemotherapy and should be considered as an influential parameter in co-culture systems. Further studies that characterize the molecular elements secreted by cells as well as signaling pathways using our platform and similar 3D co-culture systems for each cancer type will be remarkably beneficial to the cancer research community.

## Conclusion

To the best of our knowledge, this study is the first attempt to address the factor of distance in the differential inhibition of tumor cell proliferation by human fibroblasts. Our impedimetric cell-based results revealed that the neighbor suppression effect of normal fibroblasts depends on not only cell type and manner of interaction but also the distance between the 2 cell lines. A549 tumor cells were intensively inhibited within 0.25 mm from MRC-5 fibroblasts, whereas no inhibition was observed at 3 mm and further. With the capability of automatic measurements, our high-throughput assay may facilitate the preliminarily evaluation of desired cell–cell interactions in different growth or treatment conditions before the utilization of any other biological assays.
